# Mitochondrial deoxyguanosine kinase is required for female fertility in mice: DGUOK is required for female fertility in mice

**DOI:** 10.3724/abbs.2024003

**Published:** 2024-02-07

**Authors:** Yake Gao, Rui Dong, Jiacong Yan, Huicheng Chen, Lei Sang, Xinyi Yao, Die Fan, Xin Wang, Xiaoyuan Zuo, Xu Zhang, Shengyu Yang, Ze Wu, Jianwei Sun

**Affiliations:** 1 Center for Life Sciences Yunnan Key Laboratory of Cell Metabolism and Diseases State Key Laboratory for Conservation and Utilization of Bio-Resources in Yunnan School of Life Sciences Yunnan University Kunming 650091 China; 2 Department of Reproductive Medicine the First People’s Hospital of Yunnan Province NHC Key Laboratory of Preconception Health Birth in Western China Kunming 650100 China; 3 Department of Cellular and Molecular Physiology The Penn State University College of Medicine Hershey PA 17033 USA

**Keywords:** DGUOK, infertility, mitochondria, nucleoside salvage pathway, oocyte maturation

## Abstract

Mitochondrial homeostasis plays a pivotal role in oocyte maturation and embryonic development. Deoxyguanosine kinase (DGUOK) is a nucleoside kinase that salvages purine nucleosides in mitochondria and is critical for mitochondrial DNA replication and homeostasis in non-proliferating cells. *Dguok* loss-of-function mutations and deletions lead to hepatocerebral mitochondrial DNA deletion syndrome. However, its potential role in reproduction remains largely unknown. In this study, we find that *Dguok* knockout results in female infertility. Mechanistically, DGUOK deficiency hinders ovarian development and oocyte maturation. Moreover, DGUOK deficiency in oocytes causes a significant reduction in mitochondrial DNA copy number and abnormal mitochondrial dynamics and impairs germinal vesicle breakdown. Only few DGUOK-deficient oocytes can extrude their first polar body during *in vitro* maturation, and these oocytes exhibit irregular chromosome arrangements and different spindle lengths. In addition, DGUOK deficiency elevates reactive oxygen species levels and accelerates oocyte apoptosis. Our findings reveal novel physiological roles for the mitochondrial nucleoside salvage pathway in oocyte maturation and implicate DGUOK as a potential marker for the diagnosis of female infertility.

## Introduction

Infertility poses a global health challenge, impacting approximately 15% of couples worldwide [1]. The process of successful reproduction involves the normal maturation of oocytes and spermatozoa, followed by fertilization, embryo development, and implantation. Several factors influence fertility, such as maternal age, oocyte quality, endometrial thickness, and sperm motility. Despite the utilization of assisted reproductive technology, many couples are unable to achieve pregnancy. Research indicates that a range of genetic factors contribute to oocyte maturation arrest, fertilization failure, embryo stasis, and pre-implantation embryo demise, all of which underlie the complexity of infertility [ 2‒ 5]. In addition, premature ovarian insufficiency is a major cause of female infertility due to early loss of ovarian function [ 6, 7]. It affects 3.7% of women younger than 40 years [8], yet its molecular etiology is still unclear [ 9‒ 11]. Therefore, it is key to detect infertility early and treat childbearing-aged women early to improve the quality of the population quality and fertility.

Oocyte maturation is a prerequisite for successful fertilization. Mammalian oocytes pass through the germinal vesicle (GV) stage and metaphase I (MI) stage to reach metaphase II (MII) [ 12, 13]. In the MII stage, oocytes extrude from the first polar body and gain the capacity to be fertilized [12]. Engagement of mature oocytes with sperm results in the generation of zygotes, which subsequently undergo cleavage and early embryonic development before implantation [14]. Oocyte maturation involves a series of nuclear and cytoplasmic maturation events [ 15, 16]. Nuclear maturation includes germinal vesicle break down (GVBD), chromosome condensation and segregation, and expulsion of polar bodies. The cytoplasmic maturation process involves reorganization of organelles, an increase in Ca ^2+^ storage, antioxidants [17], and the storage of mRNAs and proteins [16].

As the main source of ATP and a variety of intermediates for cell metabolism [ 18‒ 21], mitochondria are also involved in many cellular processes, including oocyte maturation, defense responses to oxidative stress [22], and programmed cell death [23]. It is dynamically regulated by fusion and fission processes [ 24, 25], and its activity is closely related to intraorganellar interactions [ 26, 27]. Importantly, the mitochondrial DNA (mtDNA) copy number dramatically expands 2000-fold during oocyte maturation, increasing from approximately 200 copies per cell in primordial germ cells to 400,000 copies in mature MII oocytes [28]. In addition to the increase in mtDNA copy number, oocyte maturation is accompanied by changes in dynamic mitochondrial distribution and microstructure, which might be related to increasing ATP demand, ATP production efficiency, and oxygen consumption [ 18, 21, 29, 30]. In immature oocytes, mitochondria are clustered around germinal vesicles. As oocytes mature, mitochondria are gradually dispersed in the cytoplasm, resulting in a uniform distribution of mitochondria in the cytoplasm of MII oocytes [ 18, 21].

The mitochondrial dNTP pool is maintained by the dNTP salvage pathway and is critical for mtDNA replication and homeostasis, especially in terminally differentiated cells (e.g., neuronal cells) and slow cycling cells [31]. Deoxyguanosine kinase (DGUOK) is a rate-limiting enzyme for the purine deoxynucleotide salvage pathway in mitochondria. In humans, DGUOK deficiency leads to hepatocerebral mtDNA deletion syndrome (MDS), which can lead to fatal infantile hepatocerebral disease in the most severe cases [32]. Although mtDNA replication and expansion are well documented during oocyte development, the role of the mitochondrial nucleotide salvage pathway in oocyte maturation and female infertility has not been previously reported. In this work, we found that deletion of *Dguok* leads to oocyte immaturity and female infertility in mice. Our results shed new light on the role of the mitochondrial nucleoside salvage pathway in oocyte development and female fertility.

## Materials and Methods

### Construction of *Dguok*-knockout mice

The *Dguok*-targeted small guide RNA consists of M-Dguok-e2be-gRNA Up (5′-TAGGCCCTGGCTCCCATGAGAT-3′), M-Dguok-E2BE-gRNA Down (5′-AAACATCTCATGGGAGCCAGGG-3′), M-Dguok-E2AF-gRNA Up (5′-TAGGCAGGAGCAATAGTCAACG-3′), and M-Dguok-e2af-GrNA Down (5′-AAACCGTTGACTATTGCTCCTG-3′). The target is located in the intron region on both sides of the second exon of *Dguok*, and the nucleotide sequences include SEQ ID NO. 1 (5′-GGCCCTGGCTCCCATGAGATGGG-3′) and SEQ ID NO. 2 (5′-GGCAGGAGCAATAGTCAACGAGG-3′). sgRNA and CAS9 mRNA (CAS9MRNA-1EA; Sigma-Aldrich, St Louis, USA) were injected into the nuclear region of fertilized eggs from C57BL/6 mice at concentrations of 0.1 g/L and 0.05 g/L, after which the embryos were transferred into the fallopian tubes of pseudopregnant mice. The female positive founder mice were mated with wild-type C57BL/6 mice to obtain positive heterozygous F1 mice. The F1 heterozygous mice were self-crossed to obtain homozygous female F2 mice. All mice were maintained in the Laboratory Animal Center of Yunnan University (Kunming, China) according to institutional guidelines. All animal procedures were approved by Institutional Animal Care and Yunnan University.

### Genotype detection by PCR

Mice were numbered 21 days after birth, and mouse toes were cut off. DNA was extracted using the Jacks Lab method ( https://jacks-lab.mit.edu/). This DNA was subsequently used as a template for PCR amplification. The amplification primers used were as follows: M- *Dguok*-F: 5′-CTCCCGCACTCAGTACTACAGCT-3′, M- *Dguok*-R: 5′-AGTCCAAGTCACAGGGTCCAATA-3′, M- *Dguok*-deletion: 5′-GCGACAGAACCTATAGCAGAGTG-3′.

### Ovulation and *in vitro* fertilization

Female *Dguok* ^*–/–*^, *Dguok* ^*+/–*^, and WT mice aged 6‒8 weeks were selected for ovulation induction. Pregnant Mare Serum Gonadotropin (P9970; Solarbio, Beijing, China) was injected at 6:00 p.m. at 5 IU per 10 g body weight, and human chorionic gonadotropin (M2530; Nanjing Aibei Biotechnology, Nanjing, China) was injected at 5 IU per 10 g body weight 48 h later. The female mice were subjected to humane endpoints 14 h after human chorionic gonadotropin injection, and the ovaries and fallopian tubes were placed in Petri dishes with M2 medium (M7167; Sigma-Aldrich). Oocytes were obtained from the ampulla of the fallopian tube and placed in G-IVF ^TM^ (10136; VitrolifeGmbH, Västra Frölunda, Sweden) for fertilization. Then, humane endpoints were used for male mice aged 8‒10 weeks with normal fertility, and the epididymides were placed in Petri dishes with M2 medium. The epididymis was punctured to release the concentrated sperm, and the sperm suspension was collected and placed in G-IVF ^TM^ medium droplets (200 μL) for 30 min. Sperms were collected from the edge of the droplets, and the concentration of sperms was calculated using a Makler counting chamber. The sperm and oocytes were co-cultured for 8 h, and the ratio of oocytes to sperm was approximately 1:2000. The fertilized eggs (containing 2 pronucleus) were collected and cultured in G-1 ^TM^ medium (10128; VitrolifeGmbH) for 48 h, after which the embryos were subsequently transferred to G-2 ^TM^ medium (10132; VitrolifeGmbH) for further culture to blastocysts. The culture conditions were 37°C, 6.0% CO _2_ volume concentration, and saturated humidity.

### *In vitro* maturation of oocytes

Oocytes in the germinal vesicle (GV) stage were collected from the follicles after 46‒48 h of pregnancy-mare serum gonadotropin injection. The oocytes in the GV stage were transplanted into mature culture media for 16‒18 h. The culture conditions were 37°C, 6% CO _2_ volume concentration, and saturated humidity. The mature culture medium was G-1 ^TM^ medium, and 0.1 IU/ mL follicle-stimulating hormone (HOR-253; ProSpec, Shanghai, China), 0.1 IU/ mL luteinizing hormone (HOR-268; ProSpec), and 1 μg/mL 17β-estradiol (E2758-250MG; Sigma-Aldrich) were added.

### Whole-mount immunofluorescence staining

Oocytes were fixed in 4% formaldehyde at room temperature for 30 min, washed three times in PBS containing 0.1% Tween-20 (PBS-T), permeabilized for 15 min in PBS supplemented with 0.2% Triton X-100 and then blocked in PBS-T supplemented with 2% BSA (Sigma-Aldrich) at room temperature for 1 h or overnight at 4°C. Primary and secondary antibodies were diluted in blocking solution, and staining was performed at room temperature for 1 h or overnight at 4°C. Washes were performed after the primary and secondary antibodies were added to PBS-T. The primary antibodies used were as follows: donkey anti-mouse alpha-Tubulin (1:500, sc-8035; Santa Cruz Biotechnologies, Santa Cruz, USA) and donkey anti-mouse Tom20 (1:500, sc-17764; Santa Cruz Biotechnologies). The secondary antibodies used were donkey anti-mouse Alexa Fluor 448 (1:500, ab150105; Abcam, Cambridge, UK) or donkey anti-mouse Alexa Fluor 594 (1:500, ab150108; Abcam). DAPI staining solution (AR1176; Boster, Wuhan, China) was added during secondary antibody incubation.

### Examination of the ROS levels and natural death rate of oocytes

Oocytes were collected after ovulation induction, and granulosa cells were removed via hyaluronidase digestion. Then, the oocytes were incubated with 5 μM Mito-SOX ^TM^ Red mitochondrial superoxide indicator (Invitrogen, Carlsbad, USA) or DCFH-DA (Beyotime, Shanghai, China) at 37°C for 30 min. The samples were washed 3 times with G-Mops ^TM^ medium (10129; VitrolifeGmbH) and immediately observed under a laser scanning confocal microscope (LSM 800; Zeiss, Oberkochen, Germany). The ROS fluorescence intensity was statistically analyzed using ZEN 2.6 software. In addition, oocytes without granulosa cells were collected and placed in M16 medium (M7292; Sigma-Aldrich) for further culture. The culture conditions were 37°C, 5% CO _2_ volume concentration, and saturated humidity. The rate of natural death of the oocytes was counted after 6, 32, 56, and 96 h, respectively.

### Electron microscopic analysis of mitochondria in oocytes

Oocytes were fixed in 2.5% glutaraldehyde at 4°C for 12 h, washed with PBS 3 times, and fixed at 4°C for 1 h in 1% osmic acid in the dark. Then, the cells were washed with ddH _2_O. The oocytes were individually wrapped in 2% agarose gel, stained with uranyl acetate at 4°C overnight, and washed three times with ddH _2_O. Subsequently, ethanol gradient dehydration and resin gradient embedding were performed. The embedded samples were sectioned with an ultra-thin microtome, and the samples were stained with uranyl acetate for 10 min, followed by wash with ddH _2_O, airtight staining with lead citrate for 5 min, and washing with ddH _2_O. After drying, multiple images of mitochondria in the oocytes were captured by electron microscopy, and the internal structures of the oocytes were analyzed.

### H&E staining of mouse ovaries

For histopathological analysis, the ovaries were excised, washed with saline, and fixed with 4% formalin. Afterward, the tissues were embedded in paraffin and cut into 7-μm-thick sections, which were stained with hematoxylin and eosin (H&E). Then, sections of each mouse ovary were observed under a microscope.

### Quantitative real-time PCR

After ovulation induction, the oocytes were collected, granulosa cells were removed with hyaluronidase solution, and single oocytes were collected into single tubes of lysis buffer (1 μL of RNase inhibitor was added to 19 μL of 0.2% (vol/vol) Triton X–100 solution). Reverse transcription and PCR preamplification were performed according to previous protocols [33]. We typically use 18 cycles for a single oocyte to obtain 10‒20 ng of amplified cDNA. The RT-PCR reaction conditions were as follows: 95°C, 30 sec; 95°C, 10 sec; and 60°C, 10 sec for 40 cycles. The mixture was as follows: 5 μL of cDNA template, 10 μL of 2× M5 Hiper SYBR Premix EsTaq (with Tli RNaseH) (MF002-05; Mei5bio, Beijing, China), 1 μL of forward primer, reverse primer, and ddH _2_O. The fluorescence signals were analyzed with a CFX96 real-time PCR detection system (Bio-Rad, Hercules, USA). Relative expression levels of target genes were calculated with the ΔCT method using *Rpl32* or gDNA *Actb* as an endogenous reference gene for internal normalization. A total of eight *Dguok* ^–/–^ mice and four wild-type mice were ovulated, and the reaction was repeated three times for a total of three independent experiments. The sequence of primers are listed in Table 1 .

**Table:** **Table 1** The sequence of primers used in this study

Primer	Sequence
Dnm1 (mouse) Q-PCR Forward	5′-AACTCTGATGCCCTCAA-3′
Dnm1 (mouse) Q-PCR Reverse	5′-CTTGTTCTCTAGCACGT-3′
Miga2 (mouse) Q-PCR Forward	5′-GAGCAGGCACTGAGTGT-3′
Miga2 (mouse) Q-PCR Reverse	5′-TTCTCAGCAAACTCCCT-3′
Miga1 (mouse) Q-PCR Forward	5′-ATCACAGTTCTCCTTGA-3′
Miga1 (mouse) Q-PCR Reverse	5′-ATCACAGACACAGCACT-3′
Rpl32 (mouse) Q-PCR Forward	5′-ACAATGTCAAGGAGCTGGAG-3′
Rpl32 (mouse) Q-PCR Reverse	5′-TTGGGATTGGTGACTCTGATG-3′
Q-PCR_mmtDNA_Dloop1_Forward	5′-AATCTACCATCCTCCGTGAAACC-3′
Q-PCR_mmtDNA_Dloop1_Reverse	5′-TCAGTTTAGCTACCCCCAAGTTTAA-3′
gDNA Actb (M) Forward	5′-GAGACCTCAACACCCCAG-3′
gDNA Actb (M) Reverse	5′-GAGCATAGCCCTCGTAGATG-3′

### Detection of oocyte and granulosa cell apoptosis

An Annexin V-EGFP cell apoptosis detection kit (Beyotime) was used to measure the level of oocyte or granulosa cell apoptosis. Oocytes obtained by stimulating mouse ovulation with hormones were cultured for 6 h or 36 h and washed with warm PBS in advance to ensure the subsequent combination of Annexin V-EGFP. The oocytes were subsequently transferred to the pre-diluted Annexin V-EGFP dye solution. After incubation in the dark at 37°C for 30 min, the cells were washed with warm 1×PBS and observed under a fluorescence microscope. Granulosa cells from the periphery of the oocytes were collected, and the apoptosis of granulosa cells was detected by the same procedure as that of oocytes.

### Detection of granulosa cell proliferation within the follicle

Wild-type and *Dguok* ^–/–^ mice were intraperitoneally injected with BrdU at a dose of 60 mg/kg, and the mice were sacrificed 6 h later. Ovaries were then extracted and fixed in 10% formalin at 4°C overnight. For histology, fixed ovaries were washed, dehydrated, and embedded in paraffin. Paraffin-embedded ovaries were serially sectioned at 4 μm. The reaction was performed with mouse anti-BrdU-FITC antibody (Cat. No. 364103; BioLegend, Beijing, China) at 4°C overnight, washed, and stained with DAPI for 20 min before fluorescence observation. The extent of granulosa cell proliferation within the follicle was determined by the presence of BrdU in randomly selected ovarian sections.

## Results

### *Dguok*-knockout female mice are infertile

**Figure 1:**
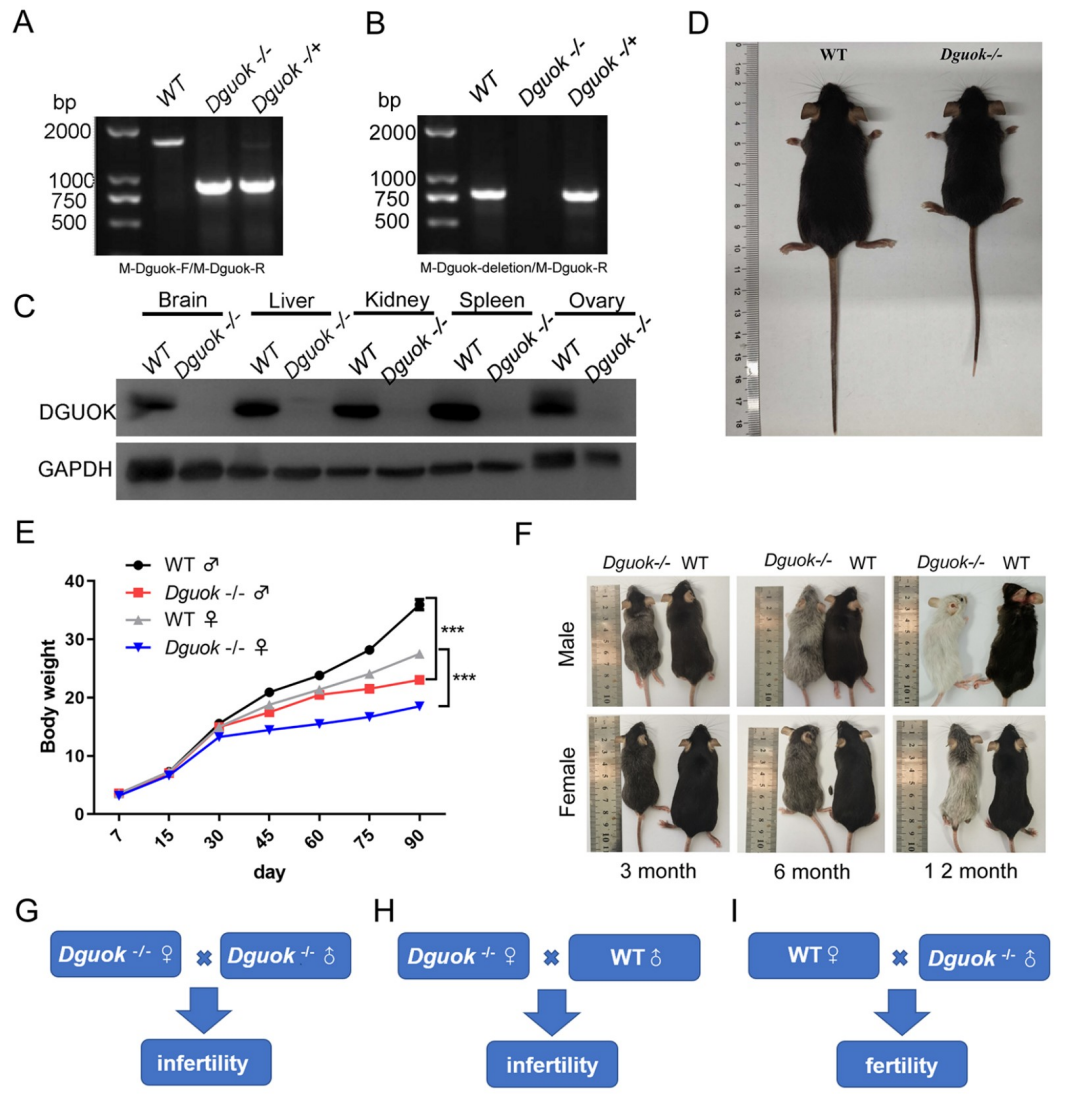
Figure 1 *Dguok* knockout induces female infertility (A) Genotyping of Dguok–/– mice with PCR primers M-Dguok-F and M-Dguok-R. (B) Genotyping of Dguok–/– mice with PCR primers M-Dguok-deletion and M-Dguok-R. (C) Western blot analysis of DGUOK expression in different tissues in WT and Dguok–/– mice; n=3. (D) Representative image of 7-week-old female WT and Dguok–/– mice. (E) Weight comparison between Dguok–/– and WT mice at different ages; n=10. ***P<0.001. (F) Representative images of Dguok–/– and WT mice at 3, 6, and 12 months. (G‒I) Schematic illustration of the breeding cage identifying the Dguok–/– female’s infertility. Each breeding scheme is equipped with 10 breeding cages.

In our previous studies, we found that DGUOK deletion affected the assembly of mitochondrial complex I and robustly inhibited lung adenocarcinoma tumor growth and metastasis and CSC self-renewal [ 34, 35]. We also found that DGUOK regulated nicotinamide adenine dinucleotide (NAD ^+^) biosynthesis independent of mitochondrial complex I [36]. To further investigate the pathophysiological function of DGUOK, we constructed *Dguok*-knockout mice ( Supplementary Figure S1A) in which the second exon of *Dguok* was completely deleted. The homozygous *Dguok*-knockout mice ( *Dguok* ^*–*/ *–*^) were validated by genotyping PCR and the absence of gene expression ( Figure 1A–C). *Dguok* ^–/–^ mice appeared normal except for a decreased body size due to modest growth retardation ( Figure 1D). The weight of *Dguok-*knockout mice was approximately 15% lower than that of control mice ( Figure 1E). These mice also exhibited a premature aging phenotype ( Figure 1F), indicating that DGUOK deficiency-mediated mitochondrial dysfunction plays a key role in aging, which is consistent with the findings of previous research [37].

Importantly, we found that mating *Dguok* ^–/–^ male mice with *Dguok* ^*–*/ *–*^ female mice produced no progeny ( Figure 1G), which suggested that DGUOK is required for fertility. Then female *Dguok* ^–/–^ mice were mated with wild-type (WT) male mice in ten cages, but did not produce any progeny ( Figure 1H), while mating between WT female mice and male *Dguok* ^–/–^ mice produced a litter size comparable to that of mating between WT C57BL/6 mice ( Figure 1I). Our results suggested that homozygous *Dguok* deletion results in female infertility but not in male mice. To investigate whether DGUOK deficiency affects mouse gender and survival, we analyzed the offspring ratio of homozygous male mice with *Dguok* deletion to heterozygous female mice. Our results showed that the proportions of male and female and the proportions of homozygous and heterozygous in the offspring obeyed Mendel’s law. We also assessed the number of offspring of *Dguok* ^–/–^ males and *Dguok* ^+/–^ females. The results showed that the model obeyed Mendel’s law ( Supplementary Figure S1B‒E). Taken together, these findings suggest that *Dguok* knockout leads to female infertility.

### DGUOK deficiency results in smaller ovaries and fewer ovulations

**Figure 2:**
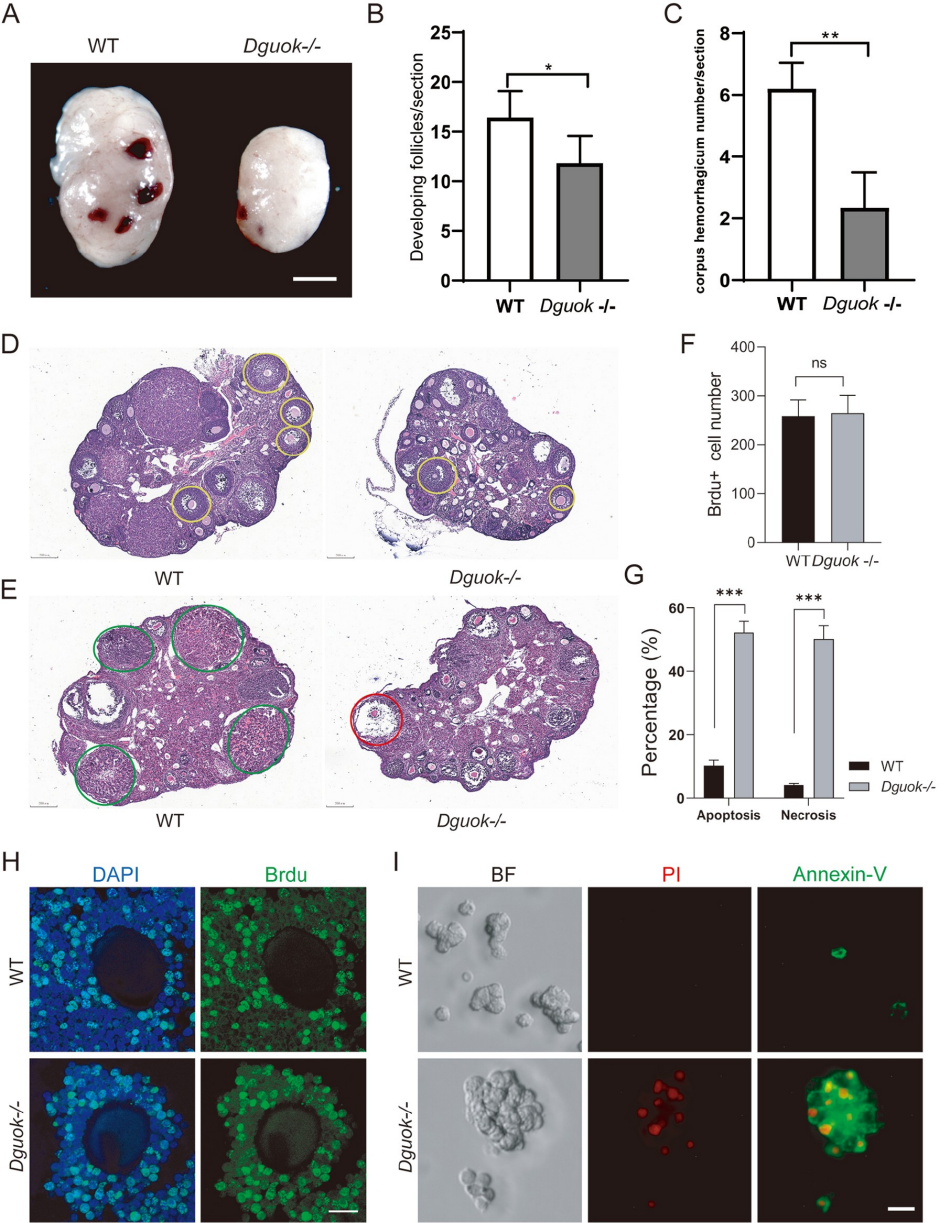
Figure 2 DGUOK deficiency results in smaller ovaries and fewer ovulations (A) Representative images of ovary morphology between WT and Dguok–/– mice. Scale bar: 1 mm. (B,C) The number of developing follicles (B) and corpus hemorrhaglcum (C) per section was counted in WT and Dguok–/– mice; n=3. *P<0.05 and **P<0.01. (D) Representative images of HE staining of the ovarian structure. Ovaries from 6-week-old WT and Dguok–/– mice were embedded, sectioned, and stained with hematoxylin and eosin (H&E). Developing follicles were indicated by yellow circle. Scale bar: 200 μm. (E) Representative images of H&E staining of ovarian structures in 6-week-old WT and Dguok–/– mice stimulated by PMSG and HCG. Corpus hemorrhagicum (green circle) from WT and anovulatorydominant follicles from Dguok–/– mice (red circle) are indicated. Scale bar: 200 μm. (F) The BrdU+ cell number of developing follicles was counted; n=3, ns (not significant) indicates P>0.05. (G) Statistical analysis of the number of apoptotic and necrotic granulosa cells in WT and Dguok–/– mice; n=3, *** P<0.001. (H) Representative immunofluorescence images of WT and Dguok–/– ovarian sections stained with BrdU-FITC antibody; n=3. Scale bar: 20 μm. (I) Representative images of Annexin-V/PI staining indicating granulosa cell apoptosis and necrosis in WT and Dguok–/– mice; n=3. Scale bar: 20 μm.

To investigate female infertility in *Dguok* ^–/–^ mice, we examined the development of ovaries and follicles and found that the ovary size was significantly smaller in *Dguok* ^–/–^ mice than in WT mice ( Figure 2A, and Supplementary Figure S2A). Histological analysis of the ovaries of 8-week-old female *Dguok*-knockout mice showed numerous immature follicles, including primordial follicles with monolayer granulosa cells ( Figure 2D). In comparison, the WT mice ovaries had more developing follicles (WT, 16.40±1.208 *vs* *Dguok* ^–/–^, 11.80±1.241) with multiple layers of granular cells ( Figure 2B,D). Follicle development retardation was also observed in the ovaries of *Dguok* ^–/–^ mice after ovulation was induced by pregnant mare’s serum gonadotrophin (PMSG) and human chorionic gonadotrophin (HCG). We found that most of the oocytes were not expelled, indicating an impaired response to hormones ( Figure 2E). In comparison, the WT mice ovaries had more corpus hemorrhagicum (WT, 6.20±0.37 *vs* *Dguok* ^–/–^, 2.33±0.67) and more excreted oocytes (WT, 26.82±1.80 *vs* *Dguok* ^–/–^, 17.27±1.46) ( Figure 2C,E, and Supplementary Figure S2C). In summary, the ovaries from *Dguok* ^–/–^ mice were hypoplastic, and the number of developing follicles and ovulations was significantly reduced ( Figure 2D, and Supplementary Figure S2B).

During follicular development, oocyte maturation mainly depends on the regulation of energy substrates and multiple signals provided by granulosa cells [ 38, 39]. Therefore, the effects of *Dguok* deletion on granulosa cell proliferation and apoptosis were also examined. We found that granulosa cell proliferation (WT, 258±19.50 *vs* *Dguok* ^–/–^, 264±21.55) within the primary or secondary follicles in *Dguok* ^*–*/ *–*^ mice was not significantly different from that in WT mice ( Figure 2F,H, and Supplementary Figure S2D,E). However, the percentage of granulosa cell apoptosis in *Dguok* ^*–*/ *–*^ mice was significantly greater than that in WT mice ( Figure 2G,I, and Supplementary Figure S2F), which explains the significant reduction in granulosa cells around oocytes in *Dguok* ^*–*/ *–*^ mice ( Supplementary Figure S2B). Taken together, our results indicate that DGUOK deficiency caused abnormal development of ovaries and oocytes.

**Figure 3:**
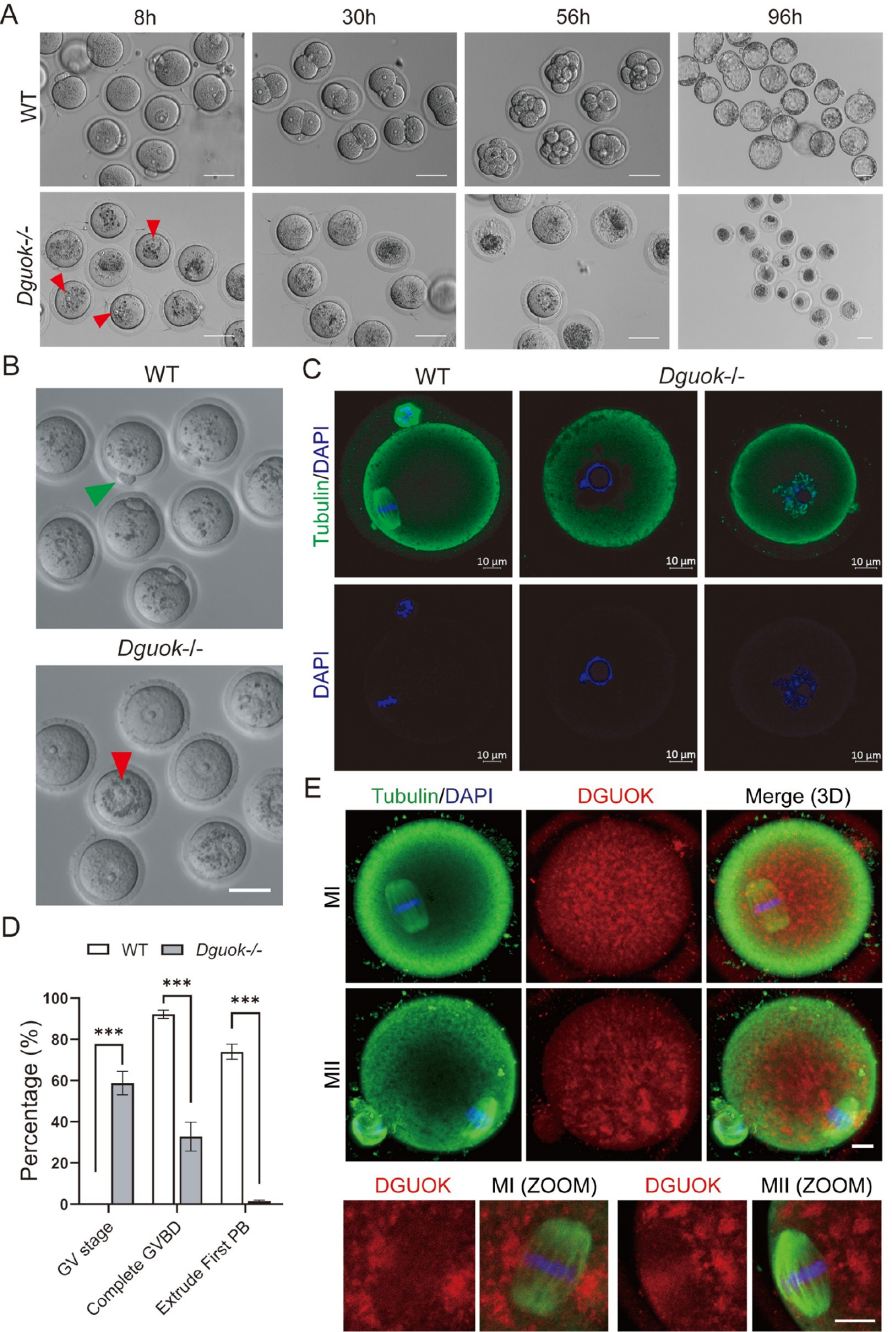
Figure 3 DGUOK regulates mouse oocyte meiotic maturation (A) Representative image of fertilization, 2-cells, 8-cells, and blastocyst formation after in vitro fertilization from WT and Dguok–/– mice; red arrows indicate GV oocytes; n=3. Scale bar: 50 μm. (B) Representative images of oocytes from WT and Dguok–/– mice. The green arrow indicates that the first polar body oocyte is expelled, red arrows indicate GV oocytes; n=3. Scale bar: 50 μm. (C) Representative images of tubulin staining in MII oocytes from WT mouse, in GV or Pre-MI oocytes from Dguok–/– mouse. Immunofluorescence staining was performed with antibodies against tubulin (green) and DAPI (blue); n=3, Scale bar: 10 μm. (D) Quantification of maturation rate in vivo of oocytes from WT and Dguok –/– mice; n=3. ***P<0.001. (E) Representative images of oocytes from WT mice for DGUOK and tubulin staining. Immunofluorescence staining was performed with antibodies against tubulin (green), DGUOK (red), and DAPI (blue); n=3. Scale bar: 10 μm.

To exclude the possibility that infertility was due to an abnormal internal fertilization environment, we performed *in vitro* fertilization. First, we found that the size of the cumulus-oocyte complexes from *Dguok* ^–/–^ mice was significantly smaller than that from WT mice ( Supplementary Figure S2B). The corona was loose, and the granulosa cells in the periphery were detached ( Supplementary Figure S2B). Moreover, *in vitro* fertilization of *Dguok* ^–/–^ oocytes was unsuccessful ( Figure 3A, and Supplementary Figure S3A). Interestingly, we found that the majority of *Dguok* ^–/–^ oocytes were arrested at the germinal vesicle (GV) stage even after 8 h of co-incubation of sperms with oocytes ( Figure 3A). Moreover, there were no significant differences in the oocyte maturation rate, fertilization rate, or embryonic development potential between *Dguok* ^+/–^ and WT mice ( Supplementary Figure S3B). Together we inferred that the infertility of the *Dguok* ^–/–^ mice was due to oocyte immaturity.

### DGUOK regulates oocyte meiotic maturation

To further investigate the effect of DGUOK on oocyte meiotic maturation, we removed the granulosa cells surrounding *Dguok* ^–/–^ oocytes and examined the spindles and chromosomes to determine their meiotic progression. Interestingly, approximately 60% of the *Dguok* ^–/–^ oocytes were blocked at the GV stage and failed to resume meiosis ( Figure 3B–D), while approximately 37% of the *Dguok* ^–/–^ oocytes completed GVBD and were blocked at prophase I ( Figure 3B–D). Only approximately 3% of *Dguok* ^–/–^ oocytes extruded their first polar body ( Figure 3D). In comparison, approximately 70% of the WT oocytes excluded the first polar body and reached metaphase II, and no oocytes were arrested at the GV stage ( Figure 3B,D). Meanwhile, we examined the distribution of DGUOK in WT oocytes. Immunofluorescence results showed that DGUOK was uniformly distributed in the cytoplasm of MI and MII oocytes but was more densely distributed around the spindle ( Figure 3E).

**Figure 4:**
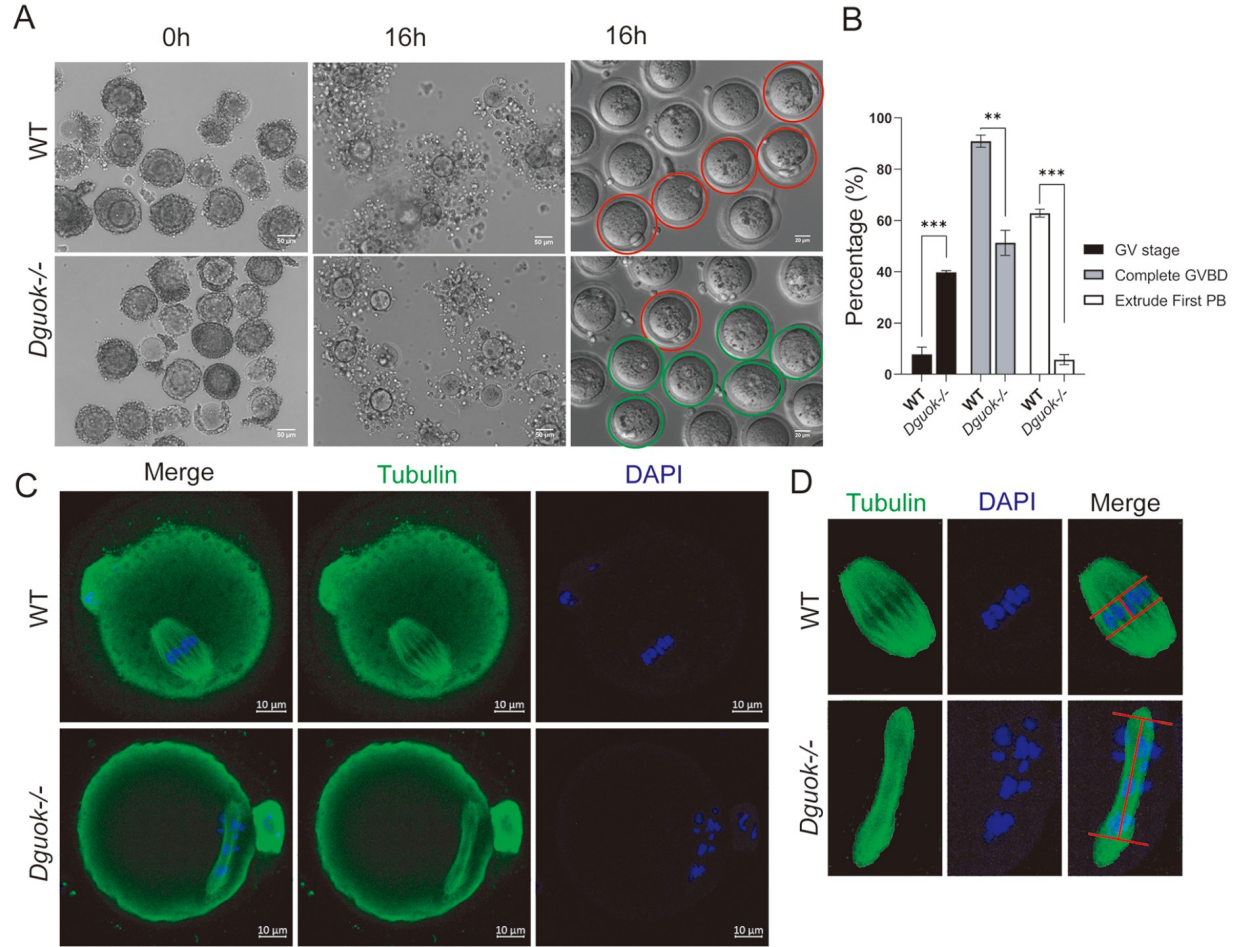
Figure 4 DGUOK deficiency results in irregular spindle structure and disordered chromosome arrangement (A) Representative images of cumulus-oocyte complexes mature in vitro (0 h, 16 h, naked-oocyte) from WT and Dguok–/– mice; the first polar body oocyte (red circle) and GV oocytes (green circle) are indicated, n=3. Scale bar: 100 μm. (B) Quantification of the GV ratio, GVBD ratio, and first pole body exclusion ratio from WT and Dguok–/– mice after 16 h in vitro maturation; n=3. **P<0.01 and ***P<0.001. (C) Representative images of tubulin staining in MII oocytes from WT and Dguok–/– mice after 16 h in vitro maturation. Immunofluorescence staining was performed with antibodies against tubulin (green) and DAPI (blue). Scale bar: 10 μm. (D) Enlarged images of oocyte spindle bodies from WT and Dguok–/– mice after 16 h in vitro maturation. The red lines represent chromosome arrangement intervals. Scale bar: 10 μm.

Furthermore, *in vitro* oocyte maturation culture was performed to investigate the effect of the intra-follicular environment on oocyte maturation. We collected oocytes at the GV stage and cultured them *in vitro* as previously described [40]. We found that after 16 h of *in vitro* maturation culture, approximately 51% of *Dguok* ^*–*/ *–*^ oocytes completed GVBD ( Figure 4B), which was significantly lower than that of the WT oocytes (~91%). Meanwhile, approximately 40% of *Dguok* ^*–*/ *–*^ oocytes remained arrested in the GV stage ( Figure 4A,B), which was significantly greater than that of the WT oocytes (~7.8%). Fortuitously, approximately 5.7% of the *Dguok* ^–/–^ oocytes extruded from the first pole body ( Figure 4A,B, and Supplementary Figure S4A), which was significantly lower than that of the WT oocytes (~63%). Importantly, even among the *Dguok* ^–/–^ oocytes with the first pole body, the structure of the spindle apparatus was irregular, and the chromosomes were not properly aligned ( Figure 4C,D, and Supplementary Figure S4B), suggesting that the *in vitro* culture environment promoted the recovery of meiosis in *Dguok* ^–/–^ oocytes to some extent. Our results revealed that DGUOK regulates mouse oocyte meiotic maturation both *in vivo* and *in vitro*.

### DGUOK is essential for mitochondrial functions in mouse oocytes

**Figure 5:**
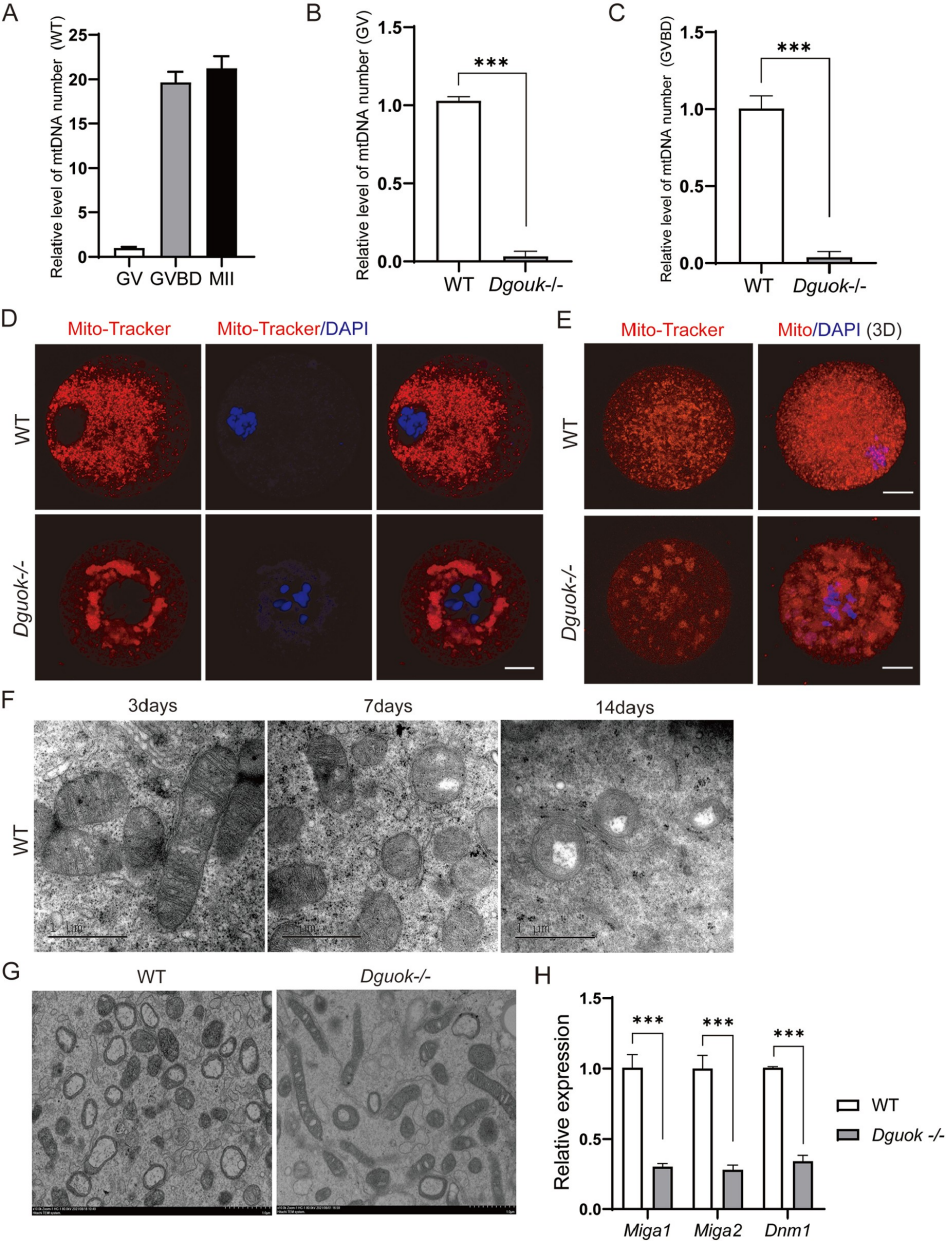
Figure 5 DGUOK is essential for mitochondrial functions in mouse oocytes (A) mtDNA content single oocytes of WT mouse at different stages, n=3. (B) Relative level of mtDNA copy number in GV oocytes from WT and Dguok–/– mice, n=3. ***P<0.001. (C) Relative level of mtDNA copy number in GVBD oocytes from WT and Dguok–/– mice, n=3. ***P<0.001. (D) Representative images of Mito-Tracker staining in WT and Dguok–/– GVBD oocytes; Scale bar: 20 μm. (E) Mito-Tracker staining in the oocytes from WT and Dguok–/– mice with PMSG and HCG stimulation. Scale bas: 20 μm. (F) TEM images of oocyte mitochondria in the ovaries of WT mice at 3, 7, and 14 days after birth. Scale bar: 1 μm. (G) Representative mitochondria TEM images of oocytes from WT and Dguok –/– mice. Scale bar: 1 μm. (H) Relative expressions of Miga1, Miga2, Dnm1 in oocytes of WT and Dguok–/– mice, n=3. ***P<0.001.

The mitochondrion harbors mtDNA which encodes 13 critical proteins for the assembly and activity of mitochondrial respiratory complexes [41]. mtDNA plays important roles during oogenesis and early embryo development [42]. Therefore, we analyzed the mtDNA copy number in *Dguok* ^–/–^ and WT oocytes. The results showed that the mtDNA copy number of the WT oocytes significantly increased from the GV stage to the MII stage ( Figure 5A). However, in *Dguok* ^*–*/ *–*^ oocytes, the mtDNA copy number was significantly lower than that in WT oocytes at both the GV and GVBD stages ( Figure 5B,C). Furthermore, we also measured the mtDNA copy number in different tissues of WT and *Dguok* ^–/–^ mice. We found that in addition to the significantly reduced mtDNA copy number in the liver, brain, kidney, and spleen of *Dguok* ^–/–^ mice, the mtDNA content in their ovaries was also severely reduced ( Supplementary Figure S5A). These results suggest that the deletion of *Dguok* severely affects mtDNA replication, which in turn affects mitochondrial function.

The distribution of mitochondria in oocytes changes dynamically with the energy demand at different stages of development. At the GV stage, mitochondria in the oocyte gradually accumulate in the perinuclear space to provide energy for nuclear rupture and spindle traction. Subsequently, mitochondria gradually distribute evenly throughout oocytes to maintain the basal metabolism level [ 21, 43, 44]. However, in *Dguok* ^*–*/ *–*^ oocytes, the movement and distribution of mitochondria were aberrant; mitochondria were irregularly clustered around the nucleus and in the cytoplasm both before and after germinal vesicle breakdown ( Figure 5D,E). Meanwhile, we also analyzed the ultrastructure of *Dguok* ^–/–^ and WT oocytes by electron microscopy. We found that mitochondria gathered in clusters in *Dguok* ^–/–^ oocytes ( Supplementary Figure S5C), which was consistent with the immunofluorescence staining results ( Figure 5D,E). Therefore, the deletion of *Dguok* leads to an imbalance in the dynamic distribution of mitochondria in oocytes, which interferes with the function of mitochondria during oocyte maturation.

It is known that mitochondria change their position and morphological structures (such as elongation, shortening, and swelling) in cells through fusion and fission to maintain mitochondrial homeostasis and normal functions [ 45, 46], which are essential for providing energy, countering stress, repairing mtDNA and mitosis [ 47‒ 49]. It has been reported that mitochondria in the oocytes of neonatal mice are mainly elongated dumbbells, but as the mice grow, the mitochondria in the oocytes become round and oval [50]. To further investigate whether deletion of *Dguok* causes abnormal changes in mitochondrial dynamics in oocytes, we analyzed the morphology of mitochondria in oocytes from *Dguok* ^–/–^ and WT mice at 3, 7, or 14 days after birth by electron microscopy. Our results showed that mitochondria were elongated dumbbells with transverse cristoids from 3-day-old WT mouse oocytes ( Figure 5F, and Supplementary Figure S5D), but they became round and vacuolar at 7 and 14 days after birth ( Figure 5F, and Supplementary Figure S5D) and until they reached adulthood (8-week-old) ( Figure 5G, and Supplementary Figure S5E). In contrast, the mitochondrial morphology of *Dguok* ^–/–^ oocytes (from 8-week-old mice) showed elongated dumbbells ( Figure 5G, Supplementary Figure S5E). Meanwhile, we examined the expressions of genes critical for the regulation of mitochondrial fission in oocytes [ 51, 52]. Similarly, the expressions of *Miga1*, *Miga2*, and *Dnm1* in *Dguok* ^–/–^ oocytes was significantly lower than those in WT oocytes ( Figure 5H). Therefore, deletion of *Dguok* impaired mitochondrial fission in oocytes. Taken together, these findings led us to conclude that DGUOK is essential for mitochondrial homeostasis and function during oocyte maturation.

### DGUOK deficiency significantly increased reactive oxygen species (ROS) and apoptosis of oocytes

**Figure 6:**
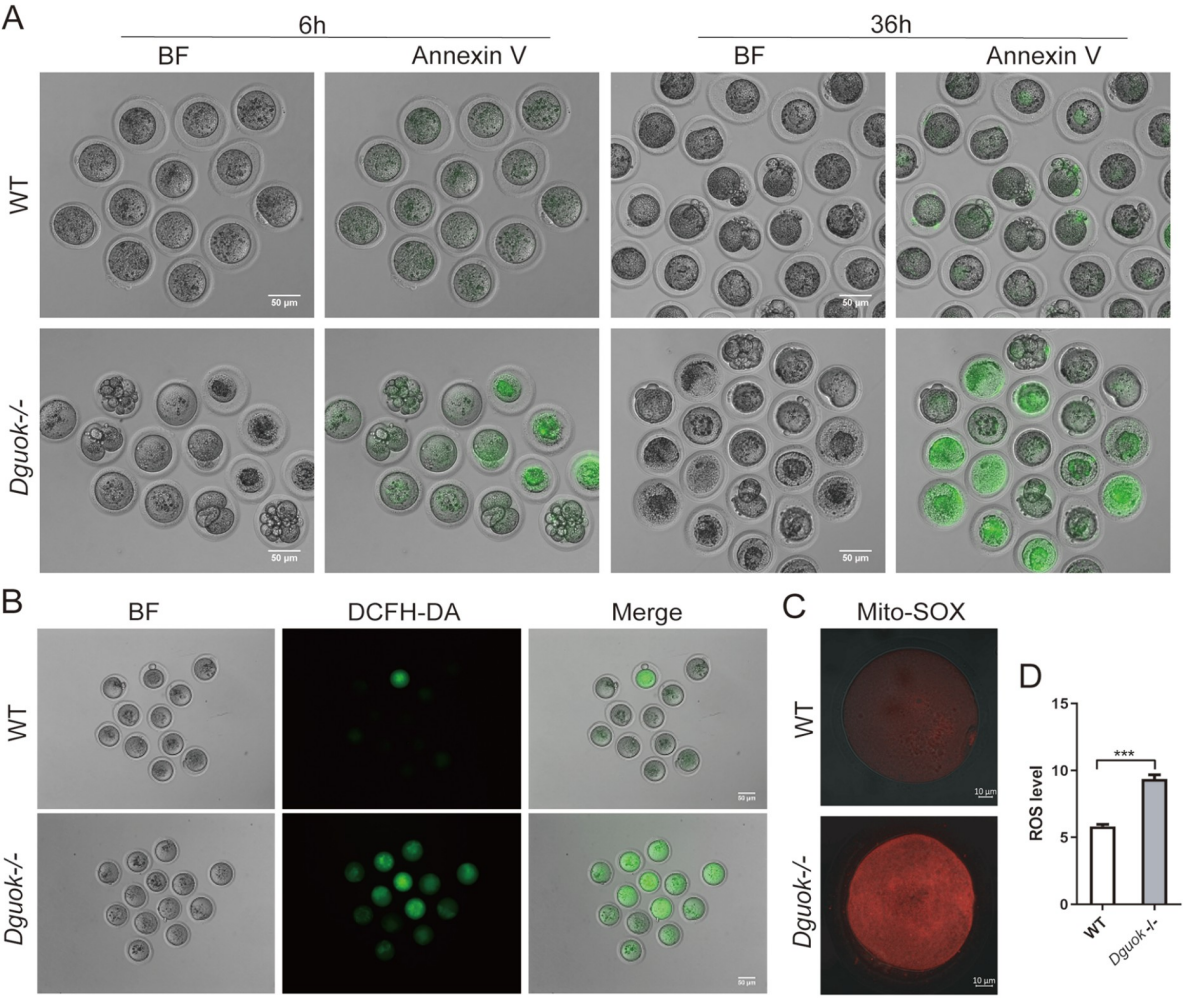
Figure 6 DGUOK deficiency significantly increases ROS levels and apoptosis of oocytes (A) Annexin-V staining in oocytes (maintained in vitro for 6 h and 36 h) from WT and Dguok–/– mice. Scale bar: 50 μm. (B) Representative image of DCFH-DA signals in oocytes from WT and Dguok –/– mice. Scale bar: 50 μm. (C,D) Representative images and quantification analysis of ROS fluorescence intensity in oocytes from WT and Dguok–/– mice, n=3. Scale bar: 10 μm. ***P<0.001.

Mitochondria are metabolic hubs and the main source of ROS. High levels of ROS cause deleterious damage to cells and may lead to apoptosis. We found that *Dguok* ^–/–^ oocytes had a greater necrosis rate than WT oocytes in *in vitro* culture medium ( Supplementary Figure S6A,B), indicating increased oocyte apoptosis in *Dguok* ^–/–^ oocytes. Subsequently, we used the Annexin-V apoptosis detection reagent to stain the oocytes maintained *in vitro* for 6 h and 36 h ( Figure 6A). As expected, the apoptosis rate of *Dguok* ^–/–^ oocytes was significantly greater than that of WT oocytes ( Figure 6A). Meanwhile, we detected ROS levels in oocytes using DCFH-DA and Mito-sox staining. Fluorescence intensity analysis revealed that the content of mtROS in *Dguok* ^–/–^ oocytes was significantly greater than that in WT oocytes ( Figure 6B–D). These findings revealed that DGUOK deficiency led to significantly increased ROS levels and increased apoptosis in oocytes.

## Discussion

In the present study, we found that DGUOK deficiency disrupts mitochondrial homeostasis and function in oocytes. Mitochondrial dysfunction causes an increase in ROS levels and oocyte apoptosis and significantly disturbs the meiotic process of oocytes, ultimately leading to oocyte immaturity. Interestingly, we found that *Dguok* deletion induces female mouse infertility but not in males. Moreover, DGUOK *-*mediated mitochondrial dysfunction plays important roles in oocyte maturation and infertility.

Oocyte maturation requires many energy-demanding processes, such as cytoskeleton remodeling, chromosome synapsis, vesicle transport, and membrane resynthesis [53]. Mitochondria are the key regulators of multiple vital cellular processes, including oocyte maturation, and provide the energy source and signal transduction for oocyte development. Mitochondrial dysfunction prevents the provision of energy and other support for these cellular processes [54]. In *Dguok* ^–/–^ mice, oocytes fail to complete meiosis, and most of them are arrested at the GV stage, indicating that mitochondria play an important role in oocyte meiosis. However, we cannot exclude the possibility that the expressions of several key mitochondrial genes are impaired in *Dguok* ^–/–^ mice, possibly leading to meiotic arrest in oocytes.

The cytoplasmic maturation of oocytes is indispensable for fertilization. Mitochondria are inherited from the maternal cytoplasm and are potential contributors to cytoplasmic maturation. The mtDNA copy number was reported to correlate with the potential of human oocytes to be fertilized [55]. It has been reported that infertility at an advanced maternal age is associated with mtDNA mutation, suggesting that mtDNA plays key roles in infertility [28] and may serve as a potential biomarker for embryo viability in assisted reproduction [ 56, 57]. Thus, the correlation between mitochondria and reproduction, particularly in oocyte maturation, is underlined, although the underlying mechanism is unclear due to the lack of tools.

In the process of follicular development, the development and maturation of oocytes depend mainly on the provision of energy substrates by granulosa cells [ 38, 39]. Meanwhile, in the process of oocyte maturation, granulosa cells transmit a variety of signals through gap junctions, which participate in the initiation of oocyte meiosis and the maturation of the oocyte cytoplasm [58]. The autocrine and paracrine substances of granulosa cells can promote the proliferation of granulosa cells and the growth of follicles. At the same time, the interaction between granulosa cells and thecal cells is important for the development and maintenance of normal follicle functions. Therefore, granulosa cells play important regulatory roles in the initiation, growth, and atresia of primordial follicles [59]. We found that the number of growing follicles in the ovaries of *Dguok* ^–/–^ mice was significantly lower than that in the ovaries of WT mice, while the number of atretic follicles was significantly greater. There were significantly fewer granulosa cells in the periphery of the oocytes than in the periphery of the WT oocytes, which may be related to the abnormal growth, differentiation, and apoptosis of granulosa cells in the *Dguok* ^–/–^ mice. Due to DGUOK deficiency, abnormal mitochondrial function in granulosa cells may lead to an abnormal follicular microenvironment, which in turn inhibits the development and maturation of oocytes. These findings may explain why *Dguok* ^*–*/ *–*^ oocytes are arrested at the GV stage *in vivo* but can partially resume meiosis in mature culture *in vitro*. In addition, the ovarian insufficiency caused by DGUOK deficiency also provides a potential molecular target for the pathogenesis of primary ovarian insufficiency (POI).

Taken together, our data support that DGUOK regulates oocyte maturation and maternal reproduction. Future investigations are needed to determine whether *Dguok* affects maternal factors. Our findings could lead to therapeutic strategies for the treatment of infertility and could lead to the development of targets for prenatal diagnosis.

## Supporting information

434Supplementary_Figures_upload

## Supplementary Data

Supplementary data is available at *Acta Biochimica et Biophysica Sinica* online.
