# Supplementary material for: Mitochondrial deoxyguanosine kinase is required for female fertility in mice: DGUOK is required for female fertility in mice
Source: Acta Biochim Biophys Sin (Shanghai). 2024 Feb 7;56(3):427–39. doi: 10.3724/abbs.2024003 (PMC10984852; doi:10.3724/abbs.2024003)
Supplement: 434Supplementary_Figures_upload [file 434Supplementary_Figures_upload.pdf]

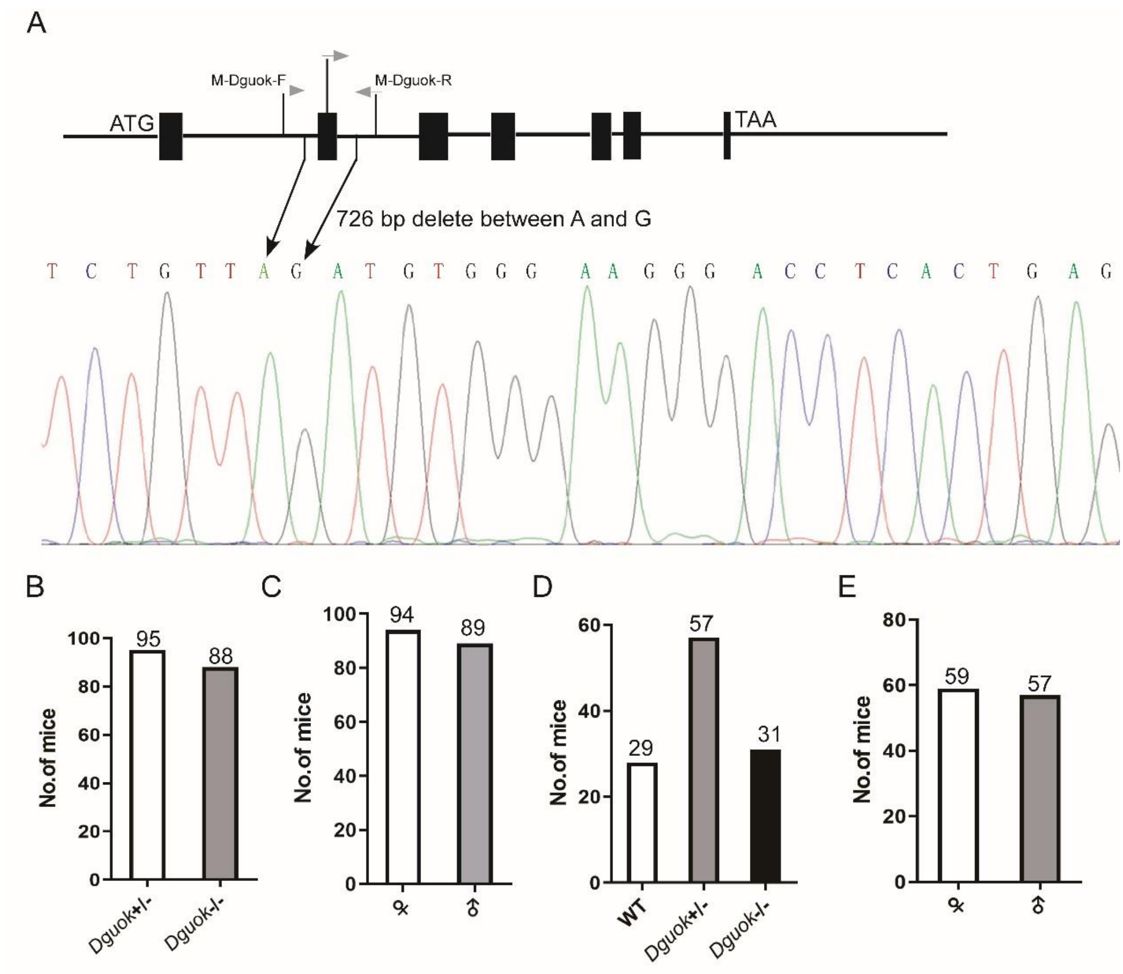

Supplementary Figure S1. Construction of *Dguok*-knockout mice (A) Scheme of 726 bp deletion in *Dguok*-knockout mice. (B) Statistical number of offspring of $Dguok^{-/-}$ male with $Dguok^{+/-}$ female. (C) Statistical number of male and female offspring of $Dguok^{-/-}$ male with $Dguok^{+/-}$ female. (D) Statistical number of wild: heterozygous: homozygous offspring of $Dguok^{+/-}$ inbred. (E) Statistical number of male and female offspring of $Dguok^{+/-}$ inbred. Each breeding scheme is equipped with 10 breeding cages.

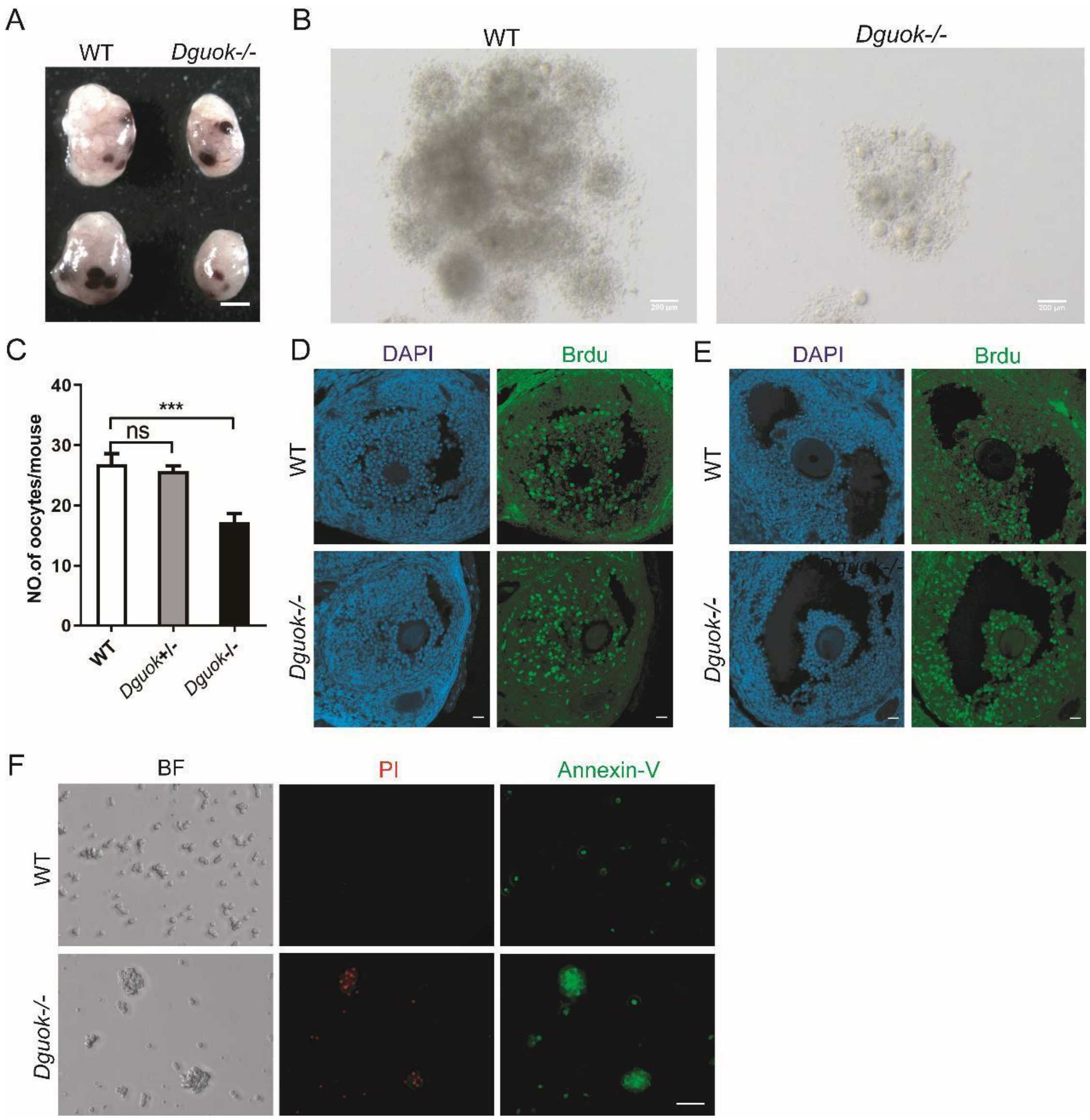

Supplementary Figure S2. DGUOK deficiency results in smaller ovaries and fewer ovulations (A) Representative images of ovary morphology between WT and *Dguok*$^{-/-}$ mice. Scale bar: 1 mm. (B) Representative images of cumulus-oocyte complexes in WT and *Dguok*$^{-/-}$ mice. Scale bar: 200 µm. (C) Statistics on the number of oocytes from WT and *Dguok*$^{-/-}$ mice. $n = 3$, ns indicates $P > 0.05$, ***$P < 0.001$. (D) Representative immunofluorescence micrographs of primary follicles from WT and *Dguok*$^{-/-}$ ovarian sections stained with BrdU-FITC antibody (green) and DAPI (blue). Scale bar: 20 µm. (E) Representative immunofluorescence micrographs of secondary follicles from WT and *Dguok*$^{-/-}$ ovarian sections stained with BrdU-FITC antibody (green) and DAPI (blue); Scale bar : 20 µm. (F) Representative images of Annexin-V/PI staining indicating granulosa cell apoptosis and necrosis in WT and *Dguok*$^{-/-}$ mice. Scale bar: 50 µm.

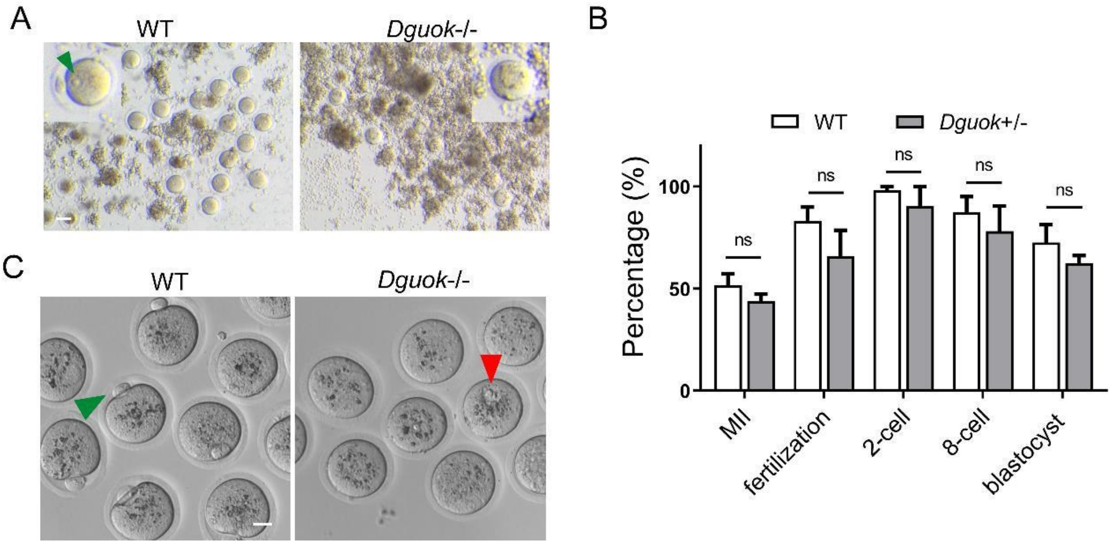

Supplementary Figure S3. DGUOK is essential for mouse oocyte meiotic maturation (A) Representative images of WT and *Dguok*$^{-/-}$ mice oocytes after *in vitro* fertilization (IVF) for 8 h. Green arrows indicate male pronucleus. *n*=3. Scale bar: 50 μm. (B) Quantification of stages in MII, fertilization, 2-cells, 8-cells, and blastocyst from WT and *Dguok*$^{+/-}$ mice oocyte after IVF. *n*=3, ns indicates *P*> 0.05. (D) Representative image of oocytes after IVF from WT and *Dguok*$^{-/-}$ mice. Green arrow indicates that the first polar body oocyte is expelled, and red arrow indicates GV oocytes. *n*=3. Scale bar: 20 μm.

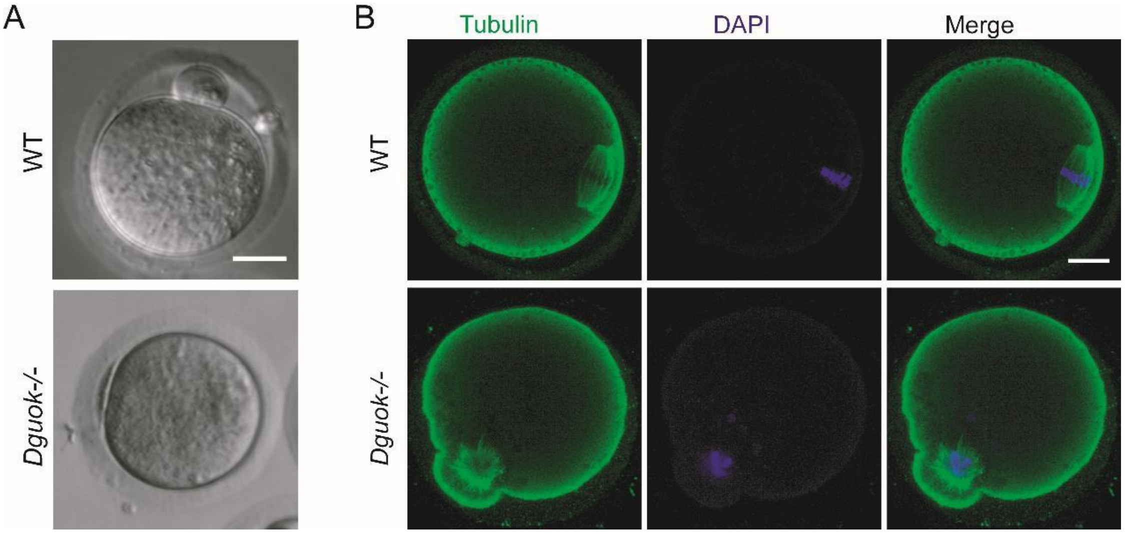

Supplementary Figure S4. DGUOK deficiency results in abnormal meiosis of oocytes (A) Representative images of WT and *Dguok*$^{-/-}$ mice oocytes after mature *in vitro*. Scale bar: 20 μm. (B) Representative image of tubulin staining in MII-like oocyte from WT and *Dguok*$^{-/-}$ mice after 16 h *in vitro* mature. Immunofluorescence with antibodies against Tubulin (green) and DAPI (blue). Scale bar: 10 μm.

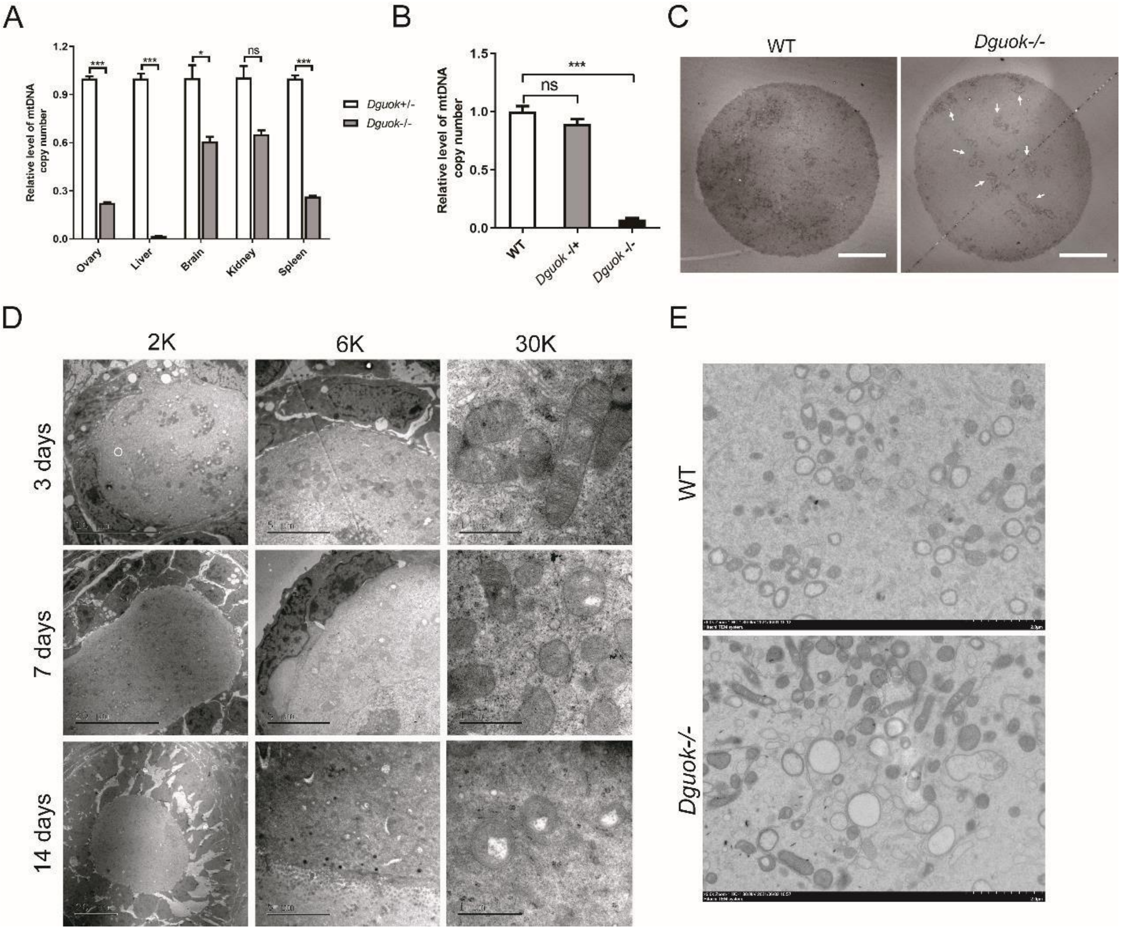

Supplementary Figure S5. DGUOK is essential for mitochondrial functions in mouse oocytes (A) Relative level of mtDNA copy number in different tissues of WT and *Dguok*$^{-/-}$ mice. *n*=3. ns indicates *P*>0.05, **P*<0.05, ****P*<0.001. (B) Relative level of mtDNA copy number in oocytes from WT, *Dguok*$^{-/+}$, and *Dguok*$^{-/-}$ mice. *n*=3. ns indicates *P*>0.05, **P*<0.05, ****P*<0.001. (C) Representative mitochondrial distribution TEM images of oocytes from WT and *Dguok*$^{-/-}$ mice. White arrows indicate mitochondrial aggregation. Scale bar: 20 μm. (D) TEM images of oocyte mitochondria in the ovaries of WT mice at 3 days, 7 days, and 14 days after birth. The magnification was 2 K, 6 K, 30 K, respectively. Scale bar: 1 μm. (E) Representative mitochondria TEM images of oocytes from WT and *Dguok*$^{-/-}$ mice. Scale bar: 1 μm.

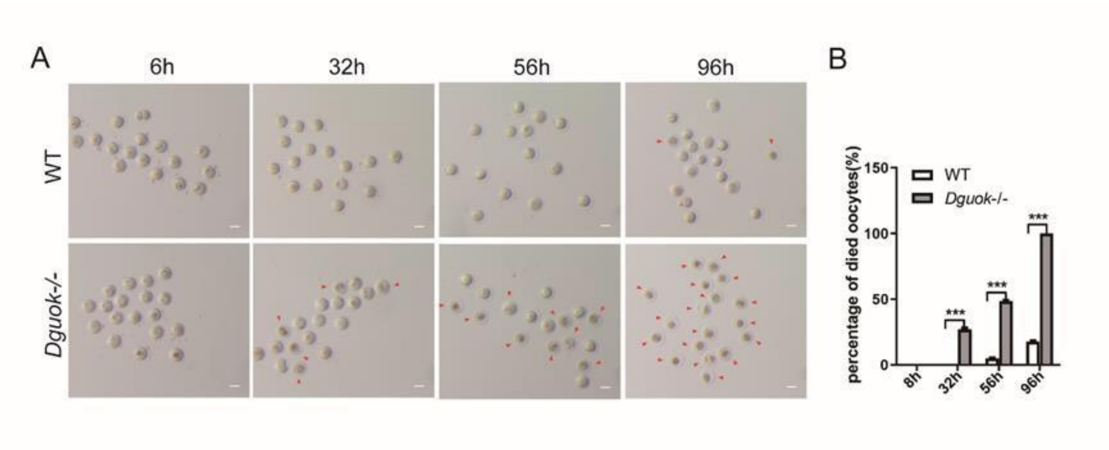

Supplementary Figure S6. DGUOK deficiency significantly increases apoptosis of oocytes (A) Natural death profile of WT and *Dguok*$^{-/-}$ mice oocytes at 6 h, 32 h, 56 h, and 96 h. Dead oocytes are indicated by red arrows. Scale bar: 50 µm. (B) Quantification of the dead oocyte ratio from WT and *Dguok*$^{-/-}$ mice. *n*=3. ****P*<0.001.
